# Supplementary material for: The Shepherd and the Hunter: A Genomic Comparison of Italian Dog Breeds
Source: Animals (Basel). 2023 Jul 27;13(15):2438. doi: 10.3390/ani13152438 (PMC10417656; doi:10.3390/ani13152438)
Supplement: Supplementary file 1 [file animals-13-02438-s001.zip › Figure S2.pdf]

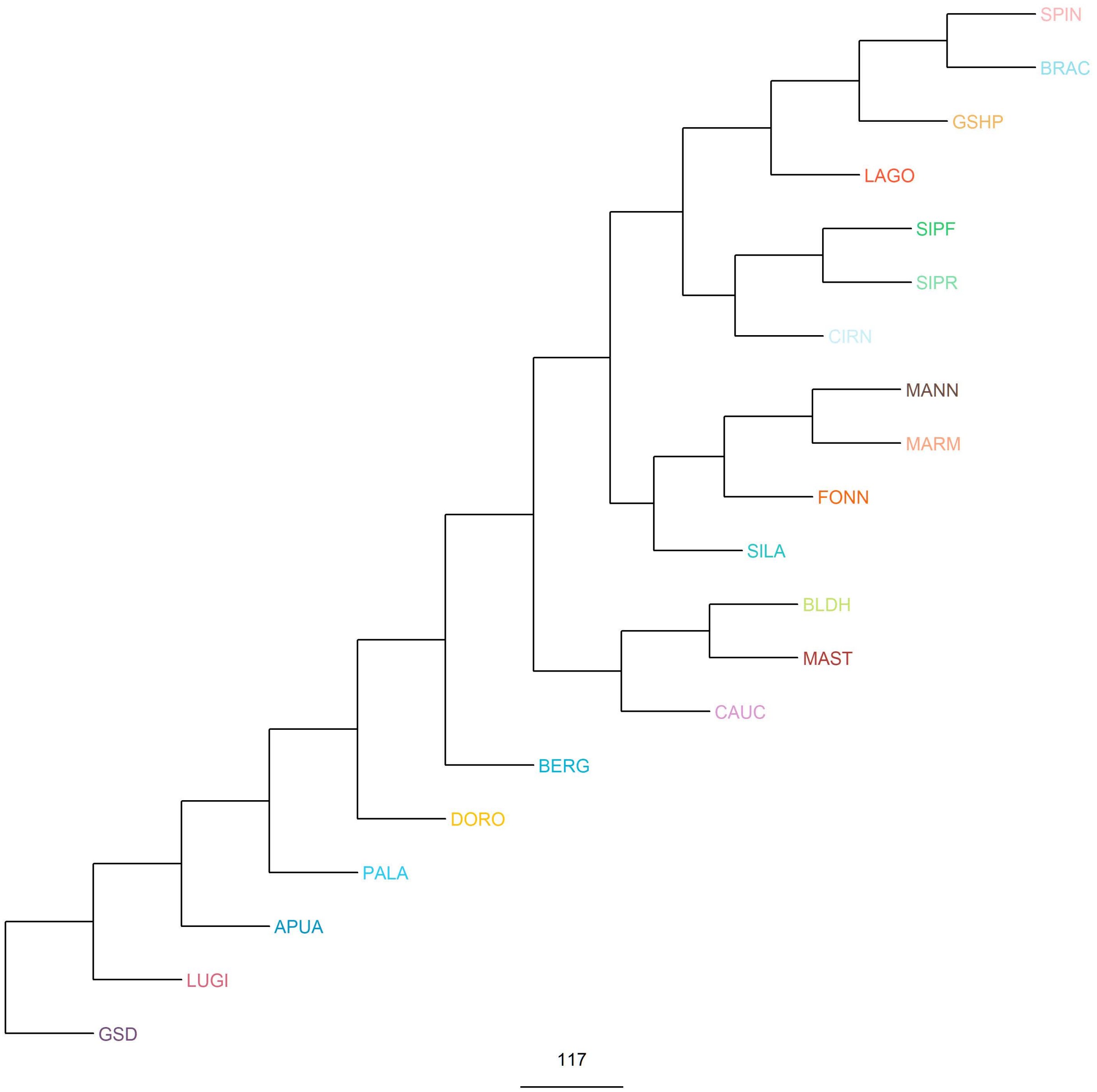


**Figure S2.** Phylogenetic tree based on Reynolds distances of Italian and non-Italian breeds. Italian hunting dogs: Bracco italiano (BRAC), Cirneco dell'Etna (CIRN), Lagotto Romagnolo (LAGO), Segugio Italiano Pelo Forte (SIPF), Segugio Italiano Pelo Raso (SIPR), and Spinone Italiano (SPIN). Italian shepherd dogs: Pastore Apuano (APUA), Bergamasco shepherd dogs (BERG), Pastore d'Oropa (DORO), Fonni's dogs (FONN), Lupino del Gigante (LUGI), Mannara dogs (MANN), Maremma and the Abruzzi sheepdogs (MARM), Pastore della Lessinia e del Lagorai (PALA), and Pastore della Sila (SILA). Non-Italian breeds: Bloodhound (BLDH), Caucasian shepherd dog (CAUC), German shepherd dog (GSD), and Mastiff (MAST).
