# Supplementary material for: The Shepherd and the Hunter: A Genomic Comparison of Italian Dog Breeds
Source: Animals (Basel). 2023 Jul 27;13(15):2438. doi: 10.3390/ani13152438 (PMC10417656; doi:10.3390/ani13152438)
Supplement: Supplementary file 1 [file animals-13-02438-s001.zip › Table S1.pdf]

Table S1. Description and pictures of the Italian breeds included in the present study. All the details come from the official breed standards.

Breed	Group	FCI group	Recognition	Coat and colour	Size	Ears
**Bracco Italiano**	Hunting dogs (gun dogs)	Group 7 (Pointing dogs) Section 1 (Continental pointing dogs, Type "Braque")	International (FCI Standard N° 202)	Short, dense, and glossy hair.<br>Colours: white; white with patches of orange or amber or brown colour; liver roan. A symmetrical facial mask is preferred.<br>Pink to flesh-coloured or brown nose.	Medium-large<br>HW: 58-67 cm for males, 55-62 cm for females.<br>Weight: 25-40 kg.	Long and drop.
**Lagotto Romagnolo**	Hunting dogs (gun dogs)	Group 8 (Retrievers - Flushing dogs - Water dogs) Section 3 (Water dogs)	International (FCI Standard N° 298)	Woolly, felted, semi-rough, and tightly curled hair, with a visible undercoat.<br>Colours: off-white solid; white with brown or orange patches; orange or brown roan; brown or orange with/without white. Brown mask and tan markings are allowed.<br>From light to dark brown nose.	Medium-small size.<br>HW: 43-48 cm for males, 41-46 cm for females.<br>Weight: 13-16 kg for males, 11-14 kg for females.	Triangular and drop.
**Spinone Italiano**	Hunting dogs (gun dogs)	Group 7 (Pointing dogs) Section 1 .3 (Continental pointing dogs, Type "Griffon")	International (FCI Standard N° 165)	4-6 cm long and rough hair, without fringes; long and stiff hair-garnish forming thick eyebrows, moustaches, and beard. No undercoat.<br>Colours: pure white; white with orange or brown markings; orange or brown (chestnut) roan with/without coloured patches. Tricolour, tan markings, and black are not permitted.<br>Pink to flesh-coloured or brown nose.	HW: 60-70 cm for males, 58-65 cm for females.<br>Weight: 32-37 kg for males, 28-30 kg for females.	Triangular and drop.
**Cirneco dell'Etna**	Hunting dogs (hounds)	Group 5 (Spitz and primitive types) Section 7 (Primitive type hunting dogs)	International (FCI Standard N° 199)	Hair with vitreous and dense texture, smooth on head, ears, and legs, 2.5 cm long, sleek, and fitting on the body and tail.<br>Colours: fawn in all shades; tan with white markings on head/chest/feet/point of tail/belly.<br>From light to dark flesh-colour to brown nose.	Medium size.<br>HW: 46-50 cm for males, 44-48 cm for females.<br>Weight: 10-13 kg for males, 8-11 kg for females.	Erect.
**Segugio Italiano a Pelo Forte**	Hunting dogs (hounds)	Group 6 (Scent hounds and related breeds) Section 1.2 (Medium-sized hounds)	International (FCI Standard N° 198)	Rough hair, not longer than 5 cm, with beard.<br>Colours: solid fawn in all shades; black and tan. White markings on muzzle/skull/chest/neck/pastern/hocks/feet/tip of the tail are admitted but not desirable in fawn dogs, whereas in black and tan dogs only a white star on the chest is admitted ("tricolour" coat).<br>Black nose.	Medium size.<br>HW: 52-58 cm for males, 48-56 cm for females.<br>Weight: 18-28 kg.	Triangular and drop, with a torsion drawing the entire leather forward.
**Segugio Italiano a Pelo Raso**	Hunting dogs (hounds)	Group 6 (Scent hounds and related breeds) Section 1.2 (Medium-sized hounds)	International (FCI Standard N° 337)	Short and smooth hair with straight horse texture.<br>Colours: solid fawn in all shades; black and tan. White markings on muzzle/skull/chest/neck/pastern/hocks/feet/tip of the tail are admitted but not desirable in fawn dogs, whereas in black and tan dogs only a white star on the chest is admitted ("tricolour" coat).<br>Black nose.	Medium size.<br>HW: 52-60 cm for males, 50-58 cm for females.<br>Weight: 20-28 kg for males, 18-26 kg for females.	Triangular and drop, with a torsion drawing the entire leather forward.

**Fonni's dog**	Shepherd dogs (livestock guardians)	Group 2 (Pinscher and Schnauzer - Molossoid and Swiss Mountain and Cattledogs)	RSA (FCI Standard N° 902)	Goat hair, with dense and woolly undercoat, forming furnishing (eyebrows and beard) and, in males, mane. Short-haired variety is admitted.<br>Colours: black, ash, or honey, which can be solid or brindled.<br>Black nose.	Medium size.<br>HW: 56-60 cm for males, 52-56 cm for females.<br>Weight: 29-35 kg for males, 25-30 kg for females.	Triangular and drop.
**Mannara dog**	Shepherd dogs (livestock guardians)	Group 2 (Pinscher and Schnauzer - Molossoid and Swiss Mountain and Cattledogs)<br>Section 2.2 (Molossoids, mountain type)	RSR (FCI Standard N° 903)	Dense, semi-long, and wavy or curly hair, forming a mane especially in males. The undercoat is present.<br>Colours: black, light liver, or tan (in different shades), all of them with/without white patches, which however should not be predominant. Tan markings (brindled or not) are admitted. Gray and brindle coats are admitted but not desirable.<br>Black, brown, or anthracite-coloured nose.	Large size.<br>HW: 69 cm (>65 cm) for males, 64 cm (>59 cm) for females.	Triangular and drop.
**Maremma and the Abruzzi sheepdog**	Shepherd dogs (livestock guardians)	Group 1 (Sheepdogs and cattle dogs except Swiss cattledogs)<br>Section 1 (Sheepdogs)	International (FCI Standard N° 201)	Long and rather harsh hair, flat lying on the body, longer around the neck and the edge of the hindquarters. Abundant undercoat in the winter.<br>Colour: solid white.<br>Black nose.	Large size.<br>HW: 67-73.5 cm for males, 62-70 cm in females.<br>Weight: 40-52 kg for males, 35-45 kg for females.	Triangular and drop.
**Pastore della Sila**	Shepherd dogs (livestock guardians)	Group 1 (Sheepdogs and cattle dogs except Swiss cattledogs)<br>Section 1 (Sheepdogs)	RSA (FCI Standard N° 904)	Hair is semi-long (>6 cm on the body), dense, forming a mane and fringes on the rear limbs; might be slightly wavy. Abundant undercoat in the winter.<br>Colours: black with/without white patches on chest/fingers/tip of the tail; black and tan, which can present a little white marking on the head; sable in different shades (from gray to tan), with/without white patches on chest/fingers/tip of the tail.<br>Black nose.	Large size.<br>HW: 60-68 cm for males, 56-64 cm for females.<br>Weight: 40-50 kg for males, 35-45 kg for females.	Triangular and drop.
**Bergamasco shepherd dog**	Shepherd dogs (herding dogs)	Group 1 (Sheepdogs and cattle dogs except Swiss cattledogs)<br>Section 1 (Sheepdogs)	International (FCI Standard N° 194)	Goat hair with wavy flocks on the front part of the body; woolly and forming broad strands, with a long and soft undercoat, from the middle of the chest to the rear.<br>Colours: solid gray or with gray patches of all shades. Tinges of Isabella and light fawn permitted. Opaque all-black colour is admitted.<br>Black nose.	Medium size.<br>HW: 60±2 cm for males, 56 ± 9 cm for females.<br>Weight: 32-38 kg for males, 26-32 kg for females.	Semi-drop.
**Lupino del Gigante**	Shepherd dogs (herding dogs)	Group 1 (Sheepdogs and cattle dogs except Swiss cattledogs)<br>Section 1 (Sheepdogs)	Not officially recognized	Long-haired variety: smooth with rough texture, fringes on rear limbs, and woolly undercoat / with beard and moustaches. Short-haired variety: short hair, with a rough texture and woolly and dense undercoat.<br>All colour combinations are admitted. Wolf sable and merle are the most diffuse varieties, but solid colour and brindle, as well as the presence of white patches, are allowed.<br>Black nose.	Medium size.<br>HW: 50-62 cm for males, 48-58 cm for females.<br>Weight: 20-30 kg.	Erect.

**Pastore Apuano**	Shepherd dogs (herding dogs)	Group 1 (Sheepdogs and cattle dogs except Swiss cattledogs) Section 1 (Sheepdogs)	RSA (FCI Standard N° 908)	Semi-long hair with thick undercoat. Colours: merle, black, black and tan, fawn, gray. Not too extended white patches are admitted. Black nose.	Medium size. HW: 60 ± 9 cm for males, 55 ± 9 cm for females.	Erect
**Pastore della Lessinia e del Lagorai**	Shepherd dogs (herding dogs)	Group 1 (Sheepdogs and cattle dogs except Swiss cattledogs) Section 1 (Sheepdogs)	RSA (FCI Standard N° 907)	Semi-long hair, with goat or smooth texture, might be wavy. The undercoat is present. Colours: solid black, brown, or tan, with a possible darker mask; blue merle; red merle. Tan markings are admitted but not desirable. White patches are admitted but not desirable if continuous and extended. The head should not be completely white. Black or brown nose, which may have pink patches.	Medium size. HW: 58 ± 5 cm for males, 54 ± 5 cm for females.	Erect or semi-erect.
**Pastore d'Oropa**	Shepherd dogs (herding dogs)	Group 1 (Sheepdogs and cattle dogs except Swiss cattledogs) Section 1 (Sheepdogs)	RSA (FCI Standard N° 905)	Short or semi-long; harsh, smooth, or woolly, with undercoat. Colours: blue merle, black and tan, tan, tricolour, black saddle, grey with white and tan markings. Not too extended white patches are admitted. Black nose.	Medium size. HW: 45-60 cm. Weight: 18-35 kg.	Erect or semi-erect.

HW: Height at withers.

Italian hunting dogs

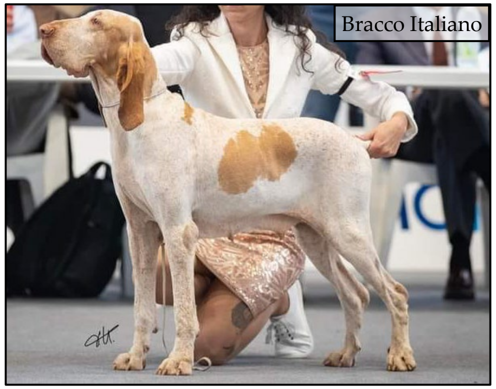
Bracco Italiano

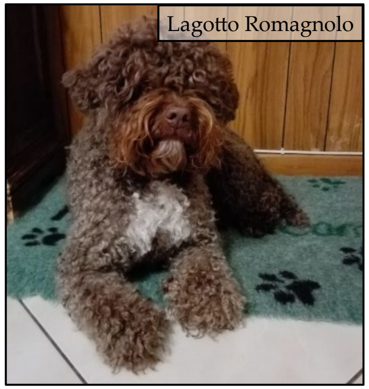
Lagotto Romagnolo

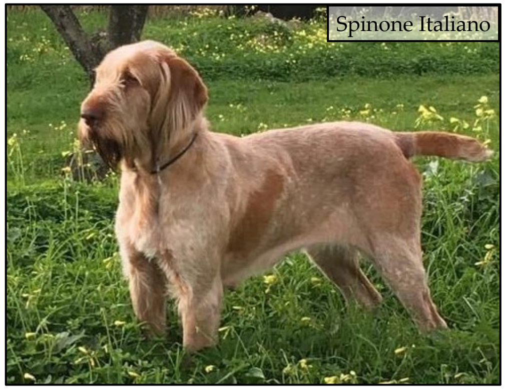
Spinone Italiano

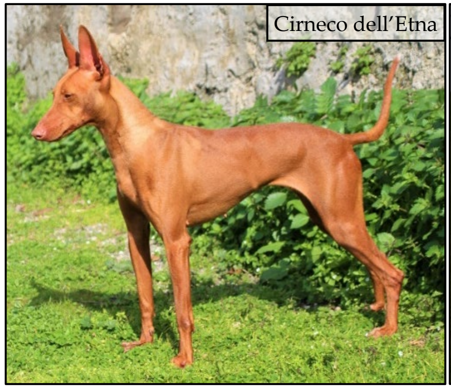
Cirneco dell’Etna

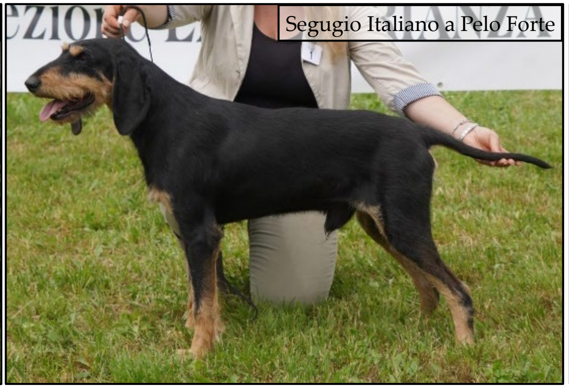
Segugio Italiano a Pelo Forte

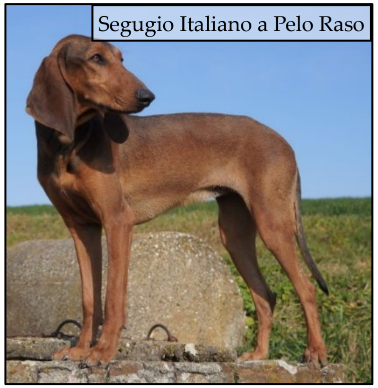
Segugio Italiano a Pelo Raso

Italian shepherd dogs

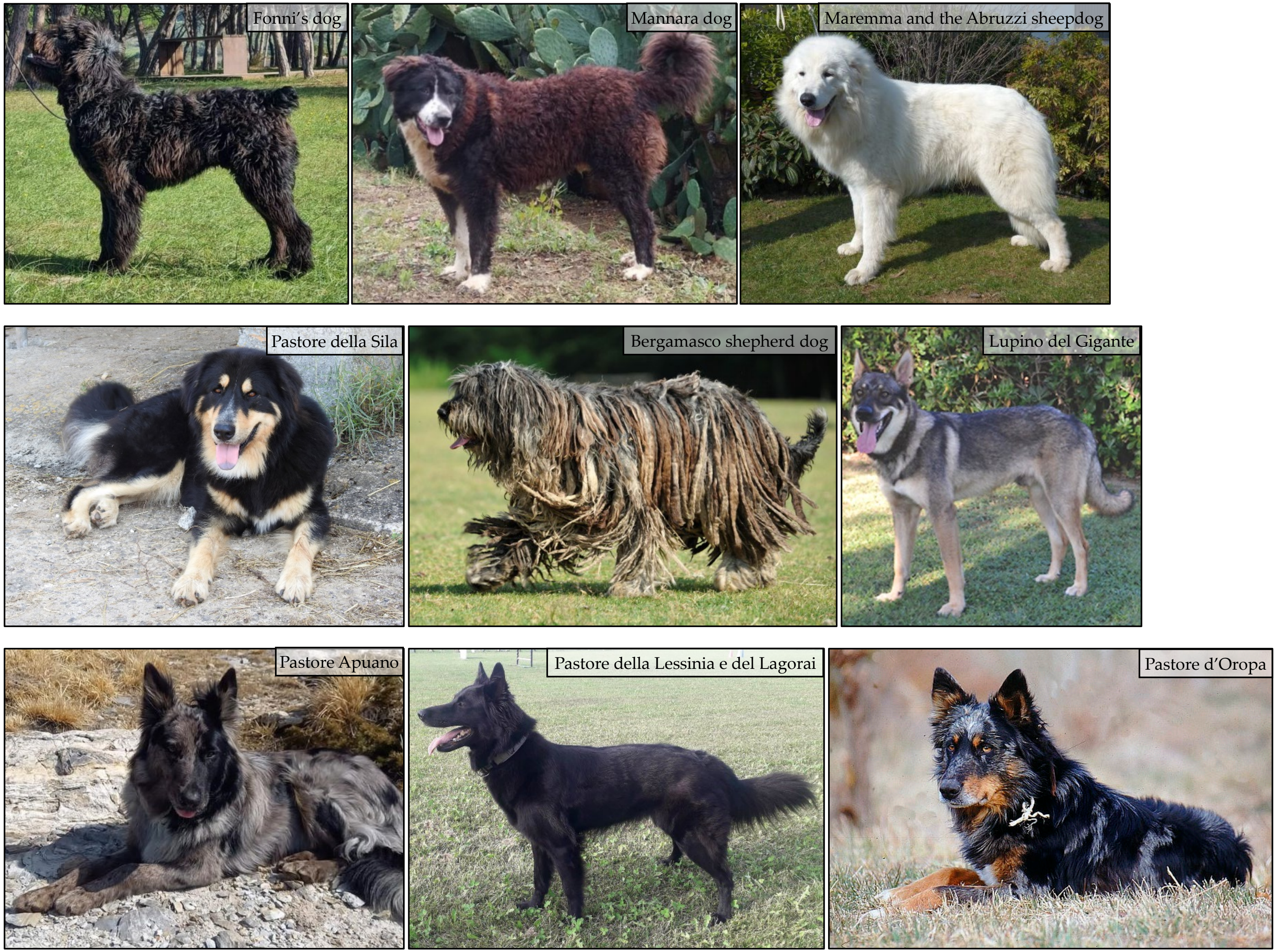

Photo credits

Bracco Italiano: Dog: Brenda, Breeder: Stefano Montesi, Owner: Giustino Di Pisa

Lagotto Romagnolo: Dog: Dante delle Gualdarie, Breeder: S. Bernardi, Owner: O. Tonelli

Spinone Italiano: Dog: Epithelium Galizia, Breeder: Cav. P. Amadori, Owner: N. Randazzo

Cirneco dell'Etna: Breeder and Owner: R. Miuccio

Segugio Italiano a Pelo Forte: Dog: Moro di Pompiano, Breeder: "All.to di Pompiano", Owner: G. Stucchi, Photographer: E. Nava

Segugio Italiano a Pelo Raso: Dog: Vicamar Beba, Breeder and Owner: "All.to Vicamar"; Photographer: E. Nava

Fonni's dog: Owner: A. Capelli

Mannara dog: Dog: Giorgino dei Tumminia, Breeder and Owner: G. Tumminia

Maremma and the Abruzzi sheepdog: Breeder: "Della Perla Maremmana", Owner: M. Donadoni

Pastore della Sila: Breeder and Owner: F. D. Scarpino / Associazione per la tutela del cane da Pastore della Sila

Bergamasco shepherd dog: Breeder: "Di Vallescrivia"; Owner: L. Guidobono Cavalchini

Lupino del Gigante: Owner: Owner: C. Ielli

Pastore Apuano: Dog: Grey, Breeder and Owner: D. Bertone

Pastore della Lessinia e del Lagorai: Owner: E. Pellizzari

Pastore d'Oropa: Owner: Associazione Amici Cane d'Oropa
